# Comparison of Preoperative Nutritional Assessment Tools for Predicting Postoperative Pulmonary Complications in Older Adults Undergoing Cardiac Surgery

**DOI:** 10.3390/nu18132211

**Published:** 2026-07-07

**Authors:** Mantana Saetang, Panalee Kittisopaporn, Thitikan Kunapaisal, Prae Plansangkate, Chanya Deekiatphaiboon, Supphamongkhon Khunakanan, Naparat Sukkriang, Surewan Srisuwan, Rinyapas Weerapachsakul

**Affiliations:** 1 Department of Anesthesiology, Faculty of Medicine, Prince of Songkla University, Songkhla 90110, Thailand; mantana.s@psu.ac.th (M.S.); panalee.k@psu.ac.th (P.K.); prae.p@psu.ac.th (P.P.); chanya.d@psu.ac.th (C.D.); surewan.c@psu.ac.th (S.S.); sutthinee.s@psu.ac.th (R.W.); 2 Department of Surgery, Faculty of Medicine, Prince of Songkla University, Songkhla 90110, Thailand; bozo.sup@gmail.com; 3 School of Medicine, Walailak University, Nakhon Si Thammarat 80160, Thailand; rheumatonask@gmail.com

**Keywords:** postoperative pulmonary complications, cardiac surgery, malnutrition, nutritional assessment, Prognostic Nutritional Index, Nutrition Alert Form, older patient, risk stratification

## Abstract

**Background/Objectives**: Postoperative pulmonary complications (PPCs) are a major source of morbidity following cardiac surgery, particularly in older adults. While malnutrition is linked to adverse outcomes, the optimal screening tool for identifying patients at risk of PPCs remains uncertain. This study compared the predictive performance of the Geriatric Nutritional Risk Index (GNRI), Mini Nutritional Assessment–Short Form (MNA-SF), Prognostic Nutritional Index (PNI), and Nutrition Alert Form (NAF) for PPCs in older adults undergoing elective cardiac surgery. **Methods**: This prospective cohort study enrolled 217 patients aged ≥ 60 years at a tertiary university hospital. Preoperative nutritional status was assessed using the GNRI, MNA-SF, PNI, and NAF. The primary outcome was PPC development during hospitalization. Predictive performance was evaluated using receiver operating characteristic (ROC) curve analysis, and multivariable logistic regression identified independent predictors. **Results**: PPCs occurred in 86 patients (39.6%). Patients who developed PPCs had significantly higher NAF scores than those who did not (median [IQR]: 7.5 [3–12] vs. 5 [2–8], *p* < 0.001), whereas GNRI, MNA-SF, and PNI scores did not differ significantly. NAF demonstrated the highest predictive performance (AUC: 0.643, 95% CI: 0.567–0.719), followed by PNI, MNA-SF, and GNRI. However, after adjusting for clinical covariates, none of the nutritional assessment tools remained independently associated with PPCs. **Conclusions**: Among the four tools evaluated, NAF showed the highest predictive performance among the evaluated nutritional assessment tools; however, its discriminative ability was modest, and none of the nutritional assessment tools remained independently associated with PPCs after multivariable adjustment. Nutritional assessment should complement, rather than replace, established clinical risk factors in perioperative risk stratification.

## 1. Introduction

The global population is aging rapidly, leading to an increasing number of older adults undergoing cardiac surgery [1]. Despite advancements in perioperative care, postoperative pulmonary complications (PPCs) such as pneumonia, prolonged mechanical ventilation, and reintubation remain among the most frequent adverse events in this population, driving up healthcare costs, prolonging hospital stays, and reducing survival [2,3]. Older adults are uniquely susceptible to PPCs because age-related physiological declines frequently coexist with sarcopenia, frailty, reduced respiratory muscle reserve and malnutrition [4]. In cardiac surgery, these vulnerabilities are severely exacerbated by cardiopulmonary bypass, systemic inflammation, and fluid shifts [5,6,7], making accurate preoperative risk stratification essential.

Malnutrition is a critical, modifiable determinant of surgical outcomes, affecting an estimated 10% to 25% of cardiac surgery patients [8,9]. Notably, it often occurs in individuals with a normal or elevated body mass index, meaning routine clinical inspection alone is insufficient to detect nutritional risk [10]. To objectively quantify this risk, several nutritional assessment tools have been developed. Among these, the Geriatric Nutritional Risk Index (GNRI) [11,12], Mini Nutritional Assessment (MNA) [13,14], Prognostic Nutritional Index (PNI) [11], and Nutrition Alert Form (NAF) [15] are widely utilized and have demonstrated prognostic value across diverse clinical settings. Specifically, a low GNRI (less than 98) has been identified as an independent predictor of acute kidney injury, systemic infections, and overall adverse outcomes after cardiac surgery [12,16]. The MNA is a well-validated screening tool tailored for older adults that successfully predicts post-surgical complications [14]. The PNI, which integrates serum albumin concentration and absolute lymphocyte count, correlates strongly with prolonged hospital stays and increased mortality rates in cardiovascular surgery [17]. And the NAF, a multidimensional nutritional screening tool originally developed and validated for hospitalized Thai patients [18] has demonstrated robust predictive performance for adverse clinical outcomes across various hospitalized cohorts [15,18].

Although these tools have shown individual prognostic promise, evidence directly comparing their predictive performance for PPCs in older cardiac surgery patients remains limited. Consequently, the optimal screening modality for this vulnerable population has not yet been established. To address this gap, this prospective cohort study aimed to compare the predictive performance of the GNRI, MNA, PNI, and NAF for PPCs in older adults undergoing elective cardiac surgery. Our findings may help identify the most clinically useful tool for perioperative risk stratification and support targeted nutritional interventions to improve postoperative respiratory outcomes.

## 2. Materials and Methods

### 2.1. Study Design and Setting

This prospective cohort study was conducted at Songklanagarind Hospital, Prince of Songkla University, Thailand, a tertiary-care university hospital and regional referral center for cardiac surgery. Consecutive older adults scheduled for elective cardiac surgery between June 2024 and April 2026 were screened for eligibility.

### 2.2. Study Participants

Patients were eligible for inclusion if they were aged 60 years or older and underwent elective on-pump cardiac surgery, including coronary artery bypass grafting (CABG), valve surgery, or combined CABG and valve procedures. Exclusion criteria comprised patients undergoing emergency cardiac surgery, as well as those undergoing minimally invasive, robotic, transcatheter, or redo cardiac procedures.

### 2.3. Ethical Considerations

This study protocol was approved by the Institutional Review Board of the Faculty of Medicine, Prince of Songkla University, Hat Yai, Thailand (approval number: REC.67–018-8–1; 21 March 2024). The study was prospectively registered at ClinicalTrials.gov (identifier: NCT06340464) on 25 March 2024. This manuscript is reported in accordance with the Strengthening the Reporting of Observational Studies in Epidemiology (STROBE) guidelines for cohort studies.

### 2.4. Preoperative Nutritional Assessment

- Preoperative nutritional status was evaluated using four distinct assessment tools within 24 h prior to surgery. Assessment questionnaires were administered by trained investigators from the anesthesiology team using standardized forms, and the assessors were blinded to all postoperative outcomes.
- GNRI [19] was calculated according to the following formula:

GNRI = [1.489 × serum albumin (g/L)] + [41.7 × (current body weight/ideal body weight)]

Ideal body weight was defined as 22 × height^2^ (m^2^).

Patients with GNRI < 91 were classified as being at nutritional risk.

- MNA-SF [20] consists of six items evaluating food intake, weight loss, mobility, psychological stress or acute illness, neuropsychological problems, and body mass index (or calf circumference when BMI is unavailable). Scores range from 0 to 14 and were categorized as follows: normal nutritional status (12–14), risk of malnutrition (8–11), or malnutrition (0–7).
- PNI [21] was calculated using the following equation:

PNI = 10 × serum albumin (g/dL) + 0.005 × total lymphocyte count (/mm^3^)

Malnutrition was defined as a PNI of less than 38.

- NAF [15,18] is a nutritional screening tool developed and validated in hospitalized Thai patients. It assesses multiple domains relevant to nutritional risk, including anthropometric status (height/arm span, body weight, body mass index, or serum albumin/total lymphocyte count when body weight is unavailable), body habitus, recent weight change, dietary intake over the preceding 2 weeks, gastrointestinal or swallowing-related symptoms persisting for more than 2 weeks, ability to access food, and the presence and severity of comorbid disease. The total NAF score categorizes patients into normal or mild malnutrition (0–5), moderate malnutrition (6–10), and severe malnutrition (11 or greater), with higher scores indicating greater nutritional risk.

### 2.5. Data Collection

Baseline demographic and clinical characteristics were prospectively collected, including age, sex, body mass index (BMI), smoking history, and relevant comorbidities (hypertension, diabetes mellitus, dyslipidemia, chronic kidney disease, chronic obstructive pulmonary disease, and heart failure). Baseline cardiac surgical risk and functional status were stratified using the EuroSCORE II and the New York Heart Association (NYHA) functional classification, respectively. Operative variables recorded included the specific surgical procedure, cardiopulmonary bypass duration, aortic cross-clamp time, total operative time, intraoperative blood product transfusion requirements, and perioperative hemodynamic parameters.

### 2.6. Outcomes

The primary objective of this study was to compare the predictive performance of GNRI, MNA-SF, PNI, and NAF for PPCs. The discriminative ability of each nutritional assessment tool was evaluated using sensitivity, specificity, receiver operating characteristic (ROC) curve analysis, and the area under the ROC curve (AUC).

### 2.7. PPCs Definitions

Postoperative pulmonary complications (PPCs) were defined as the occurrence of one or more of the following clinical events during hospitalization:

Pneumonia: Diagnosed by the presence of compatible respiratory signs and symptoms, a new infiltrate on chest radiography, and supportive clinical findings (e.g., fever, leukocytosis, or purulent sputum).

Prolonged Mechanical Ventilation: Defined as invasive mechanical ventilation extending beyond 48 h post-surgery.

Reintubation: Defined as unplanned endotracheal reintubation occurring within 72 h of initial extubation.

Pleural Effusion Requiring Drainage: Confirmed by postoperative chest radiography demonstrating blunting of the costophrenic angle, loss of the sharp silhouette of the ipsilateral hemidiaphragm in the upright position, or displacement of adjacent anatomical structures. In the supine position, it was identified by a hazy opacity involving one hemithorax with preserved vascular markings.

Atelectasis: Radiographic evidence of postoperative lung collapse or lobar/segmental atelectasis documented in the medical record.

Pulmonary Embolism: Confirmed by targeted diagnostic imaging studies and recorded in the medical record.

Phrenic Nerve Injury: Clinically suspected diaphragmatic dysfunction, confirmed via imaging or fluoroscopic evaluation, and documented in the medical record.

Acute Respiratory Distress Syndrome (ARDS): Diagnosed according to the established Berlin criteria during hospitalization.

### 2.8. Sample Size Calculation

The sample size was estimated for receiver operating characteristic (ROC) analysis to evaluate the discriminatory performance of the nutritional assessment tools for postoperative pulmonary complications (PPCs). The calculation was performed using the online sample size calculator available at RiskCalc.org (Prediction Model: Area under ROC curve; https://riskcalc.org/samplesize/ accessed on 1 April 2024). Because no previous study had directly compared GNRI, MNA-SF, PNI, and NAF for predicting PPCs in older adults undergoing cardiac surgery, the sample size was estimated based on a null hypothesis AUC of 0.50, an anticipated AUC of 0.65, an expected PPC incidence of 30% based on prior literature [22], a two-sided alpha level of 0.05, and 90% power. A minimum sample size of 173 patients was required. To account for an estimated 10% rate of attrition or incomplete data, the final target enrollment was increased to a minimum of 191 participants.

### 2.9. Statistical Analysis

Continuous variables were presented as mean ± standard deviation (SD) for normally distributed data or as median (interquartile range [IQR]) for skewed distributions. Categorical variables were expressed as frequencies and percentages. Baseline characteristics and operative variables between patients who developed PPCs and those who did not were compared using Student’s *t*-test or the Mann–Whitney U test for continuous data, and the chi-square test or Fisher’s exact test for categorical data. The discriminative performance of the GNRI, MNA-SF, PNI, and NAF for predicting PPCs was evaluated using receiver operating characteristic (ROC) curve analysis. Areas under the ROC curve (AUCs) with 95% confidence intervals (CIs) were calculated and compared using the DeLong method. Optimal cutoff values were determined using the Youden index to compute corresponding sensitivities, specificities, positive predictive values, negative predictive values, and likelihood ratios. Calibration performance was rigorously assessed using the Brier score, the Hosmer–Lemeshow goodness-of-fit test, calibration mean absolute error (MAE), and calibration mean squared error (MSE). Univariable logistic regression analysis was initially performed to identify factors associated with PPCs. Variables with a *p*-value less than 0.20 in univariable analysis, alongside clinically relevant covariates, were entered into multivariable logistic regression models. To reduce the risk of model overfitting, the total number of candidate predictors was restricted based on the number of observed PPC events, adhering to standard events-per-variable principles. Multicollinearity among independent variables was monitored using variance inflation factors (VIFs), with VIF values less than 5 indicating the absence of problematic collinearity. Adjusted odds ratios (aORs) with 95% CIs were reported. Patients missing essential data required for nutritional screening or outcome evaluation were excluded prior to analysis. Among the 217 patients in the final analytic cohort, there were zero missing data points across nutritional scores, laboratory values, clinical covariates, or postoperative pulmonary outcomes; thus, a complete-case analysis was performed without the need for data imputation. All statistical analyses were performed using R software (version 4.6.0; R Foundation for Statistical Computing, Vienna, Austria). A two-sided *p*-value less than 0.05 was considered statistically significant.

## 3. Results

### 3.1. Patient Characteristics

**Figure 1:**
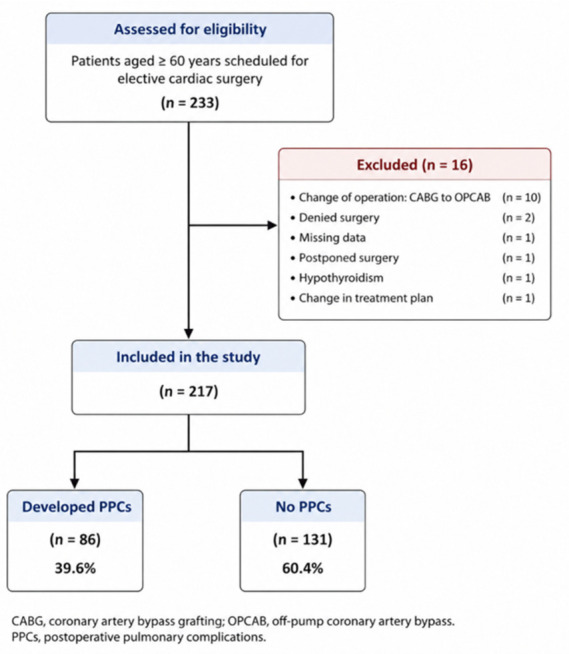
Flow diagram of patient enrollment and study inclusion.

A total of 217 older adults undergoing elective cardiac surgery were included in the study (Figure 1).

Postoperative pulmonary complications (PPCs) occurred in 86 patients (39.6%) (Table 1). Among the 86 patients who developed PPCs, pleural effusion was the most common complication, occurring in 57 patients (66.3%), followed by atelectasis in 55 patients (64.0%).

Patients who developed PPCs had a significantly higher prevalence of heart failure (51.2% vs. 33.1%, *p* = 0.013), hypertension (81.7% vs. 66.2%, *p* = 0.021), dyslipidemia (87.8% vs. 71.5%, *p* = 0.009), diabetes mellitus (48.8% vs. 32.3%, *p* = 0.024), and dialysis dependence (8.5% vs. 0.8%, *p* = 0.006) compared with those without PPCs (Table 2). The PPC group also had significantly higher EuroSCORE II values (median (IQR) 2.3 (1.2, 4.1) vs. 1.5 (1, 2.8), *p* = 0.004) and NYHA functional class (median (IQR) 2 (1, 2) vs. 1 (1, 2), *p* = 0.024). In addition, patients with PPCs demonstrated lower hemoglobin concentrations (median (IQR) 12.1 (10.7, 13) vs. 12.5 (11.1, 13.9) g/dL, *p* = 0.021), lower eGFR (median (IQR) 66 (41, 79) vs. 71 (54, 83.5)) mL/min/1.73 m^2^, *p* = 0.045), and lower lymphocyte counts (median (IQR) 1567 (1119.1, 2239.6) vs. 1917 (1536.4, 2245.5) cells/mm^3^, *p* = 0.002) (Table 2).

### 3.2. Intraoperative Characteristics

Patients who developed PPCs had significantly higher vasoactive–inotropic scores (VIS) immediately after cardiopulmonary bypass weaning (*p* = 0.029) and required a greater number of platelet units transfused (*p* < 0.001). No significant differences were observed in the type of surgery, cardiopulmonary bypass time, aortic cross-clamp time, or other transfusion requirements (Table 3).

### 3.3. Nutritional Status According to Postoperative Pulmonary Complications

The median GNRI, MNA-SF, and PNI scores were lower among patients who developed PPCs; however, these differences did not reach statistical significance (Table 4). In contrast, NAF score was significantly higher in patients who developed PPCs than in those without PPCs (median (IQR) 7.5 (3, 12) vs. 5.0 (2, 8), *p* < 0.001). When categorized according to predefined cutoff values, only NAF demonstrated a significant association with PPCs. Patients with NAF ≥ 6 had a significantly higher incidence of PPCs than those with NAF < 6 (50.5% vs. 28.7%, *p* = 0.002). No significant associations were observed for GNRI < 91 (*p* = 0.517), MNA-SF ≤ 11 (*p* = 0.730), or PNI < 38 (*p* = 0.052).

### 3.4. Predictive Performance of Nutritional Assessment Tools

**Figure 2:**
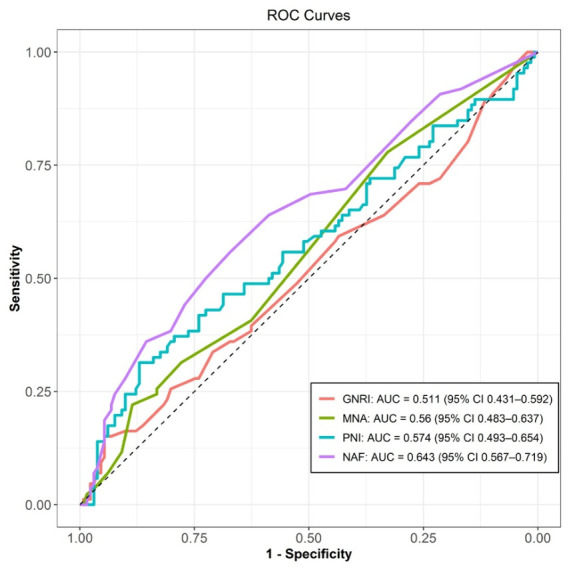
Receiver operating characteristic (ROC) curves of the Geriatric Nutritional Risk Index (GNRI), Mini Nutritional Assessment–Short Form (MNA-SF), Prognostic Nutritional Index (PNI), and Nutrition Alert Form (NAF) for predicting postoperative pulmonary complications (PPCs) in older adults undergoing cardiac surgery. Areas under the ROC curve (AUCs) with 95% confidence intervals were calculated to assess discriminative performance, and pairwise comparisons between ROC curves were performed using DeLong’s test. Among the evaluated tools, NAF showed the highest, although modest discriminative performance.

ROC analysis demonstrated that NAF had the highest discriminative performance for predicting PPCs, with an AUC of 0.643 (95% CI 0.567–0.719), followed by PNI (AUC 0.574, 95% CI 0.493–0.654), MNA-SF (AUC 0.562, 95% CI 0.486–0.639), and GNRI (AUC 0.511, 95% CI 0.431–0.592) (Table 5, Figure 2). Although NAF showed the highest AUC among the evaluated tools, its discriminative ability remained modest. Using the optimal cutoff value of 7, NAF demonstrated a sensitivity of 55.8%, specificity of 67.2%, positive predictive value of 0.527, and negative predictive value of 0.698. In comparison, the other nutritional assessment tools showed limited discriminative performance, with AUCs ranging from 0.511 to 0.574.

Pairwise comparisons of ROC curves using DeLong’s test demonstrated that the predictive performance of PNI (AUC 0.574) was significantly greater than that of GNRI (AUC 0.511; *p* = 0.027). Similarly, NAF (AUC 0.643) showed significantly better discriminative ability than GNRI (*p* = 0.002). No significant differences were observed between GNRI and MNA-SF (*p* = 0.226), MNA-SF and PNI (*p* = 0.804), MNA-SF and NAF (*p* = 0.058), or PNI and NAF (*p* = 0.108).

Calibration performance is presented in Table 6. All four nutritional assessment tools demonstrated acceptable calibration, with non-significant Hosmer–Lemeshow goodness-of-fit tests (all *p* > 0.05). Among the evaluated tools, NAF showed the best overall calibration, as reflected by the lowest Brier score (0.225), calibration mean absolute error (MAE, 0.023), and calibration mean squared error (MSE, 0.00108). PNI demonstrated the highest MAE (0.042), whereas GNRI showed the highest Brier score (0.239), indicating comparatively poorer calibration.

### 3.5. Logistic Regression Analysis for Predictors of PPCs

The results of the univariable and multivariable logistic regression analyses are presented in Table 7. VIF analysis showed that all independent variables had VIF values < 5, indicating no evidence of problematic multicollinearity among the variables included in the multivariable model.

In the multivariable analysis, atrial fibrillation (adjusted OR 2.62, 95% CI 1.06–6.76; *p* = 0.040), smaller calf circumference (adjusted OR 0.87, 95% CI 0.77–0.97; *p* = 0.023), combined CABG and valvular surgery (adjusted OR 3.37, 95% CI 1.30–9.05; *p* = 0.013), hypertension (adjusted OR 2.69, 95% CI 1.18–6.49; *p* = 0.022), and dyslipidemia (adjusted OR 3.19, 95% CI 1.31–8.42; *p* = 0.014) were independently associated with PPCs. In contrast, GNRI, PNI, MNA-SF, and NAF were not independently associated with PPCs after multivariable adjustment.

### 3.6. Postoperative Outcomes

Patients with PPCs experienced poorer postoperative outcomes, including longer ICU stays (median (IQR) 5 (3, 7) vs. 4 (3, 5) days, *p* < 0.001) and longer hospital stays (median (IQR) 14 (10, 25.2) vs. 10 (8, 14) days, *p* < 0.001) (Table 3). However, the duration of mechanical ventilation and 7-day mortality were not significantly different (Table 3). No 30-day mortality was observed in either group.

## 4. Discussion

In this prospective cohort study of older adults undergoing elective cardiac surgery, 39.6% of patients developed PPCs. Although this incidence falls within the range reported in previous studies [22,23,24], it should be interpreted cautiously because our composite PPC definition included common radiographic findings, particularly pleural effusion and atelectasis, which were the most frequent events in our cohort. Routine postoperative chest radiography may also have increased the detection of these abnormalities. PPCs remain among the most common postoperative complications and are associated with prolonged hospitalization, increased healthcare utilization, and worse clinical outcomes [22,25,26]. Older patients are particularly vulnerable because of age-related declines in pulmonary reserve, impaired immune function, frailty, and a higher burden of cardiovascular comorbidities [4]. Consistent with these observations, patients who developed PPCs in our cohort had a higher prevalence of hypertension, diabetes mellitus, dyslipidemia, heart failure, and dialysis dependence, as well as higher EuroSCORE II values.

The present study further demonstrated that nutritional status may contribute to the risk of PPCs in older cardiac surgical patients. Among the four nutritional assessment tools evaluated, NAF demonstrated the highest discriminative performance and the most favorable calibration. Nevertheless, its discriminative ability remained modest, and none of the nutritional assessment tools remained independently associated with PPCs after adjustment for clinical covariates. These findings suggest that nutritional assessment alone is insufficient for accurately predicting PPCs and should be considered a complementary component of perioperative risk stratification rather than a standalone prediction tool. This observation is consistent with previous evidence indicating that no single nutritional index fully captures perioperative risk [19,27,28,29].

The observed differences in predictive performance among the nutritional assessment tools may be explained by the distinct domains assessed by each instrument. NAF incorporates multiple components of nutritional risk, including nutritional intake, weight changes, functional status, anthropometric measurements, and clinical factors [30], which may better reflect the multifactorial nature of nutritional vulnerability in older adults. This broader assessment may explain its superior discriminative performance compared with GNRI, MNA-SF, and PNI. Calibration analysis further demonstrated that NAF had the lowest Brier score, mean absolute error, and mean squared error, indicating the most favorable calibration among the evaluated tools. However, the modest discrimination observed in the present study indicates that nutritional screening tools alone cannot adequately identify patients at high risk for PPCs and should therefore be interpreted alongside established clinical risk factors.

An important finding of the present study was that none of the nutritional assessment tools remained independently associated with PPCs after adjustment for clinical covariates. Although NAF demonstrated the highest discriminative performance and the most favorable calibration, its predictive ability was attenuated after adjustment for established clinical risk factors. This discrepancy between ROC analysis and multivariable regression highlights that a tool with the highest discriminative performance does not necessarily remain an independent predictor after adjustment for other clinically relevant variables. The relatively poor performance of GNRI was somewhat unexpected, as previous studies have reported associations between low GNRI and adverse outcomes after cardiac surgery [12,16]. One possible explanation is that GNRI primarily reflects body weight and serum albumin and may therefore be less sensitive to the complex physiological changes associated with aging, frailty, and inflammation. Similarly, although MNA-SF is a well-validated screening tool for malnutrition in older adults [14], its relatively limited discriminative ability in our cohort may limit its usefulness for identifying patients at risk of PPCs before cardiac surgery.

In addition to nutritional factors [31], several clinical variables were independently associated with PPCs, including atrial fibrillation, hypertension, dyslipidemia, combined CABG and valvular surgery, and smaller calf circumference. These findings are consistent with previous studies demonstrating that PPCs are influenced by a combination of cardiovascular comorbidity, surgical complexity, and impaired physiological reserve [32,33,34,35]. Atrial fibrillation may contribute to perioperative hemodynamic instability and pulmonary congestion [36], whereas patients undergoing combined CABG and valvular surgery are exposed to more complex procedures and greater physiological stress [35,37]. Smaller calf circumference, a surrogate marker of reduced muscle mass [38], may reflect sarcopenia and impaired respiratory muscle reserve, thereby increasing susceptibility to postoperative pulmonary complications [39]. Collectively, these findings suggest that the risk of PPCs is multifactorial and that nutritional assessment should be integrated with established clinical risk factors to improve perioperative risk stratification.

Patients who developed PPCs experienced significantly longer ICU and hospital stays [40], emphasizing the substantial clinical and economic burden associated with these complications. Although nutritional assessment alone demonstrated limited predictive value, integrating nutritional assessment with established clinical risk factors may improve perioperative risk stratification. Early identification of high-risk patients through nutritional and clinical assessment may facilitate targeted perioperative interventions, including nutritional optimization, pulmonary rehabilitation, and enhanced postoperative monitoring.

### Strengths and Limitations

A major strength of this study is its prospective design, which minimized recall and selection biases. Furthermore, we utilized highly standardized definitions for PPCs and conducted a rigorous, head-to-head comparison of four distinct, validated nutritional assessment tools. Despite these strengths, several limitations warrant consideration: First, this study was conducted at a single tertiary academic medical center, which may limit the generalizability of our findings to other geographical regions, community hospitals, or vastly different patient populations. Second, while our sample size met the minimum requirement dictated by power calculations, it may have limited our statistical power to detect subtle, small differences in discriminative performance between the four nutritional tools. Third, the routine use of postoperative chest radiography in all patients likely heightened the detection of subclinical or asymptomatic radiographic complications, particularly mild pleural effusions and focal atelectasis. Consequently, surveillance bias cannot be entirely excluded, and this intensive monitoring likely contributed to the relatively high baseline incidence of PPCs (39.6%) observed in our cohort. Fourth, although the NAF demonstrated the highest relative predictive performance, it is critical to note that all four nutritional tools exhibited only modest overall discriminative capacity (AUC less than 0.70). Finally, PPCs represent a highly heterogeneous group of clinical events driven by diverse pathophysiological mechanisms. Attempting to predict such a broad spectrum of conditions using single-domain baseline screening tools may inherently attenuate predictive performance. Future multi-center studies are needed to evaluate whether integrating multidimensional nutritional assessments with objective frailty measures, established cardiac risk scores (such as the EuroSCORE II), and intraoperative clinical variables can yield superior predictive precision for PPCs in older adults undergoing cardiac surgery.

## 5. Conclusions

Among the four nutritional assessment tools, NAF showed the highest predictive performance for PPCs. However, its discriminative ability was modest, and none of the nutritional assessment tools remained independently associated with postoperative pulmonary complications after multivariable adjustment. Therefore, preoperative nutritional assessment should be considered a complementary component of perioperative risk stratification rather than a standalone predictive tool.

## Figures and Tables

**Table 1:** Frequency of individual postoperative pulmonary complication (PPC) components among patients with PPCs.

Postoperative Pulmonary Complication Component	Patients with PPCs (*n* = 86)
Pleural effusion	57 (66.3)
Atelectasis	55 (64.0)
Reintubation within 72 h after extubation	6 (7.0)
Prolonged mechanical ventilation > 48 h	5 (5.8)
Pneumonia	3 (3.5)
Pulmonary embolism	0
Phrenic nerve injury	0
Acute respiratory distress syndrome	0

Abbreviations: h, hours; PPC, postoperative pulmonary complications.

**Table 2:** Baseline characteristics according to postoperative pulmonary complications (PPCs).

Demographic Characteristics	PPC (*n* = 86)	No PPC (*n* = 131)	*p*-Value
Age, years, median (IQR)	68 (64, 73)	68 (63, 72)	0.298
Male sex, *n* (%)	49 (57)	89 (67.9)	0.134
BMI, kg/m^2^, median (IQR)	23 (21.2, 25.7)	23.2 (20.9, 25.4)	0.852
Smoking history, *n* (%)	0 (0)	7 (5.4)	0.045
**Comorbidities**, *n* (%)	82 (95.3)	130 (99.2)	0.082
Hypertension	67 (81.7)	86 (66.2)	0.021
Diabetes mellitus	40 (48.8)	42 (32.3)	0.024
Dyslipidemia	72 (87.8)	93 (71.5)	0.009
Heart failure	42 (51.2)	43 (33.1)	0.013
CKD	23 (28)	22 (16.9)	0.079
Dialysis	7 (8.5)	1 (0.8)	0.006
Previous stroke or TIA	16 (19.5)	14 (10.8)	0.115
**Preoperative assessment**			
EuroSCORE II, median (IQR)	2.3 (1.2, 4.1)	1.5 (1, 2.8)	0.004
NYHA class, median (IQR)	2 (1, 2)	1 (1, 2)	0.024
ASA physical status, *n* (%)			0.176
3	65 (75.6)	110 (84)	
4	21 (24.4)	21 (16)	
**Laboratory variables**			
Hemoglobin (g/dL), median (IQR)	12.1 (10.7, 13)	12.5 (11.1, 13.9)	0.021
eGFR (mL/min/1.73 m^2^), median (IQR)	66 (41, 79)	71 (54, 83.5)	0.045
Lymphocyte count (/mm^3^), median (IQR)	1567.2 (1119.1, 2239.6)	1917 (1536.4, 2245.5)	0.002
Serum albumin, median (IQR)	4 (3.6, 4.4)	4.1 (3.9, 4.3)	0.284
**Anthropometric Measurements**			
Waist circumference, cm, mean (SD)	88 (11.7)	85.7 (12.7)	0.192
Hip circumference, cm, median (IQR)	92 (86, 96)	93 (88, 97)	0.384
Mid-arm circumference, cm, median (IQR)	26 (24, 28)	26 (24, 28)	0.849
Calf circumference, cm, median (IQR)	32 (29, 33.9)	32 (30, 34)	0.054
Tricep skin fold by caliper, cm, median (IQR)	2.2 (1.3, 3)	2 (1.1, 3)	0.591
**Nutritional assessment**			
GNRI, median (IQR)	101 (94.2, 106)	101 (95.5, 104.2)	0.779
MNA-SF, median (IQR)	12 (9.4, 13)	13 (11, 14)	0.115
PNI, median (IQR)	49.3 (43.6, 53.2)	50.3 (46.8, 54.1)	0.067
NAF, median (IQR)	7.5 (3, 12)	5 (2, 8)	<0.001

Abbreviations: ASA, American Society of Anesthesiologists; BMI, body mass index; CKD, chronic kidney disease; eGFR, estimated glomerular filtration rate; EuroSCORE II, European System for Cardiac Operative Risk Evaluation II; GNRI, Geriatric Nutritional Risk Index; IQR, interquartile range; MNA-SF, Mini Nutritional Assessment–Short Form; NAF, Nutrition Alert Form; NYHA, New York Heart Association; PNI, Prognostic Nutritional Index; PPC, postoperative pulmonary complication; SD, standard deviation; TIA, transient ischemic attack.

**Table 3:** Intraoperative Characteristics and Early Postoperative Outcomes According to Postoperative Pulmonary Complications (PPCs).

Variable	PPC (*n* = 86)	Non-PPC (*n* = 131)	*p*-Value
**Type of cardiac surgery, *n* (%)**			0.061
CABG	38 (44.2)	74 (56.5)	
Valve surgery	30 (34.9)	44 (33.6)	
Combined CABG + valve	17 (19.8)	13 (9.9)	
Aortic surgery	1 (1.2)	0 (0)	
**Cardiopulmonary bypass time, min, median (IQR)**	134.5 (103, 175.8)	129 (95.5, 159)	0.374
**Aortic cross-clamp time, min, median (IQR)**	79.5 (62, 121.2)	79 (54, 108.5)	0.204
**VIS**, median (IQR)			
VIS after successful CPB weaning	15 (6, 33)	10 (5, 20)	0.029
VIS at 30 min after CPB weaning	12.2 (4, 29.6)	8 (4, 18)	0.080
VIS at 60 min after CPB weaning	10 (5, 24)	7 (3.1, 15)	0.052
VIS at 90 min after CPB weaning	10 (5, 27.5)	7 (4, 15)	0.075
VIS at 120 min after CPB weaning	16 (5, 30.5)	8 (5, 25)	0.469
VIS at 150 min after CPB weaning	14.3 ± 16.2	26.6 ± 15.5	0.327
**Intraoperative blood transfusion**			
PRC transfusion, *n* (%)	77 (89.5)	110 (84.0)	0.337
PRC, units transfused, median (IQR)	2 (1, 3)	2 (1, 3)	0.604
FFP units transfused, median (IQR)	3 (3, 5)	3 (3, 5)	0.212
Platelet transfusion, *n* (%)	69 (80.2)	108 (83.1)	0.725
Platelet units transfused, median (IQR)	6 (6, 6)	6 (6, 6)	<0.001
Cryoprecipitate transfusion, *n* (%)	8 (9.4)	6 (4.7)	0.273
Duration of mechanical ventilation, hrs, median (IQR)	15 (10.2, 18)	13 (6, 16)	0.063
Days in ICU, days, median (IQR)	5 (3, 7)	4 (3, 5)	<0.001
Days in hospital stay, days, median (IQR)	14 (10, 25.2)	10 (8, 14)	<0.001
7-day mortality, *n* (%)	2 (2.3)	0 (0)	0.08

Abbreviations: CABG, coronary artery bypass grafting; CPB, cardiopulmonary bypass; FFP, fresh frozen plasma; ICU, intensive care unit; IQR, interquartile range;; PPC, postoperative pulmonary complication; PRC, packed red blood cells; VIS, vasoactive–inotropic score.

**Table 4:** Comparison of nutritional assessment scores between patients with and without postoperative pulmonary complications (PPCs).

Nutritional Assessment	PPC (*n* = 86)	Non-PPC (*n* = 131)	*p*-Value
**GNRI**			0.517
GNRI < 91, *n* (%)	14 (16.3)	16 (12.2)	
GNRI ≥ 91, *n* (%)	72 (83.7)	115 (87.8)	
GNRI score, median (IQR)	101 (94.2, 106.0)	101 (95.5, 104.2)	0.779
**MNA-SF**			0.730
MNA-SF ≤ 11, *n* (%)	35 (40.7)	49 (37.4)	
MNA-SF > 11, *n* (%)	51 (59.3)	82 (62.6)	
MNA-SF score, median (IQR)	12 (9.4, 13.0)	13 (11.0, 14.0)	0.115
**PNI**			0.052
PNI < 38, *n* (%)	10 (11.6)	5 (3.8)	
PNI ≥ 38, *n* (%)	76 (88.4)	126 (96.2)	
PNI score, median (IQR)	49.3 (43.6, 53.2)	50.3 (46.8, 54.1)	0.067
**NAF**			0.002
NAF ≥ 6, *n* (%)	55 (64.0)	54 (41.2)	
NAF < 6, *n* (%)	31 (36)	77 (58.8)	
NAF score, median (IQR)	7.5 (3.0, 12.0)	5.0 (2.0, 8.0)	<0.001

Abbreviations: GNRI, Geriatric Nutritional Risk Index; IQR, interquartile range; MNA-SF, Mini Nutritional Assessment–Short Form; NAF, Nutrition Alert Form; PNI, Prognostic Nutritional Index.

**Table 5:** ROC analysis of nutritional scores for predicting postoperative pulmonary complications.

Score	AUC	95% CI	Cutoff	Sensitivity (%)	Specificity (%)	PPV	NPV
GNRI	0.511	0.431–0.592	89.35	15.1	94.7	0.650	0.629
MNA-SF	0.562	0.486–0.639	13.00	77.9	32.8	0.432	0.694
PNI	0.574	0.493–0.654	44.46	31.4	87.0	0.614	0.659
NAF	0.643	0.567–0.719	7.00	55.8	67.2	0.527	0.698

Abbreviations: AUC, area under the receiver operating characteristic curve; CI, confidence interval; GNRI, Geriatric Nutritional Risk Index; MNA-SF, Mini Nutritional Assessment–Short Form; NAF, Nutrition Alert Form; NPV, negative predictive value; PNI, Prognostic Nutritional Index; PPV, positive predictive value.

**Table 6:** Calibration performance of GNRI, MNA-SF, PNI, and NAF for predicting postoperative pulmonary complications.

Score	Brier Score	Hosmer–Lemeshow χ^2^	*p*-Value	Calibration MAE	Calibration MSE
GNRI	0.239	9.06 (*df* = 8)	0.337	0.034	0.00182
MNA-SF	0.237	3.13 (*df* = 4)	0.536	0.034	0.00182
PNI	0.235	9.96 (*df* = 8)	0.268	0.042	0.00266
NAF	0.225	6.53 (*df* = 7)	0.479	0.023	0.00108

Abbreviations: *df*, degrees of freedom; GNRI, Geriatric Nutritional Risk Index; MAE, mean absolute error; MNA-SF, Mini Nutritional Assessment–Short Form; MSE, mean squared error; NAF, Nutrition Alert Form; PNI, Prognostic Nutritional Index.

**Table 7:** Univariable and multivariable logistic regression analyses for predictors of postoperative pulmonary complications.

	Univariate		Multivariate	
Variable	Crude OR (95% CI)	*p*-Value	Adjusted OR (95% CI)	*p*-Value
Atrial fibrillation	1.44 (0.71–2.88)	0.310	2.62 (1.06–6.76)	0.04
Serum creatinine	1.36 (1.07–1.87)	0.025	1.26 (0.99–1.71)	0.087
Waist circumference	1.02 (0.99–1.04)	0.201	1.03 (1.0–1.07)	0.072
Calf circumference	0.93 (0.86–0.99)	0.036	0.87 (0.77–0.97)	0.023
GNRI	0.997 (0.970–1.027)	0.833	1.06 (0.99–1.15)	0.096
MNA-SF	0.92 (0.83–1.02)	0.11	0.97 (0.82–1.13)	0.659
PNI	0.96 (0.92–1.00)	0.060	0.94 (0.86–1.02)	0.127
NAF	1.09 (1.04–1.15)	<0.001	1.05 (0.98–1.12)	0.167
Type of operation (ref. = CABG)				0.046
Valvular surgery	1.21 (0.64–2.26)	0.552	1.32 (0.58–2.99)	0.498
Combined CABG and valvular surgery	2.62 (1.15–6.06)	0.022	3.37 (1.3–9.05)	0.013
Hypertension	2.45 (1.26–4.98)	0.010	2.69 (1.18–6.49)	0.022
Dyslipidemia	2.82 (1.36–6.35)	0.008	3.19 (1.31–8.42)	0.014

Abbreviations: CI, confidence interval; GNRI, Geriatric Nutritional Risk Index; MNA-SF, Mini Nutritional Assessment–Short Form; NAF, Nutrition Alert Form; OR, odds ratio; PNI, Prognostic Nutritional Index.

## Data Availability

The original contributions presented in this study are included in the article.

## Abbreviations

The following abbreviations are used in this manuscript:

AUC	Area under the curve
CI	Confidence interval
GNRI	Geriatric Nutritional Risk Index
MNA-SF	Mini Nutritional Assessment–Short Form
PNI	Prognostic Nutritional Index
NAF	Nutrition Alert Form
PPC	Postoperative pulmonary complication
PPV	Positive predictive value
NPV	Negative predictive value
EuroSCORE II	European System for Cardiac Operative Risk Evaluation II
NYHA	New York Heart Association
ASA	American Society of Anesthesiologists
eGFR	Estimated Glomerular Filtration Rate
